# Comparative study on the catalytic activity of Fe-doped ZrO_2_ nanoparticles without significant toxicity through chemical treatment under various pH conditions

**DOI:** 10.1038/s41598-019-47443-7

**Published:** 2019-07-29

**Authors:** Hye-in Song, Jung A. Hong, Hangil Lee, Kwang-il Lim

**Affiliations:** 1 Department of Chemical and Biological Engineering, Sookmyung Women’s University, Seoul, 04310 Republic of Korea; 2 Department of Chemistry, Sookmyung Women’s University, Seoul, 04310 Republic of Korea

**Keywords:** Catalysis, Biocatalysis

## Abstract

Despite advances in the construction of catalysts based on metal oxide nanoparticles (MO NPs) for various industrial, biomedical, and daily-life applications, the biosafety concerns about these NPs still remain. Recently, the need to analyze and improve the safety of MO NPs along with attempts to enhance their catalytic performance has been strongly perceived. Here, we prepared multiple variants of Fe-doped zirconium oxide (Fe@ZrO_2_) NPs under different pH conditions; then, we assessed their toxicity and finally screened the variant that exhibited the best catalytic performance. To assess the NP toxicity, the prepared NPs were introduced into three types of human cells originally obtained from different body parts likely to be most affected by NPs (skin, lung, and kidney). Experimental results from conventional cellular toxicity assays including recently available live-cell imaging indicated that none of the variants exerted severe negative effects on the viability of the human cells and most NPs were intracellular localized outside of nucleus, by which severe genotoxicity is unexpected. In contrast, Fe@ZrO_2_ NPs synthesized under a basic condition (pH = 13.0), exhibited the highest catalytic activities for three different reactions; each was biochemical (*L*-cysteine oxidation) or photochemical one (4-chlorophenol degradation and OH radical formation with benzoic acid). This study demonstrates that catalytic Fe@ZrO_2_ NPs with enhanced activities and modest or insignificant toxicity can be effectively developed and further suggests a potential for the use of these particles in conventional chemical reactions as well as in recently emerging biomedical and daily-life nanotechnology applications.

## Introduction

For several decades, metal oxide nanoparticles (MO NPs) have been used as catalysts in conventional chemical reactions and next in electrochemical reactions and photoreactions^1–4^. In addition, the inherent catalytic activities of these NPs have been recently utilized in biomedical applications such as the boosting of cellular biochemical reactions, sensing of biomolecules of interest, and facilitation of protein folding^5–7^. Versatile MO NPs such as TiO_2_, SnO_2_, ZnO, and CuO NPs are used for applications in various scientific and engineering fields^8–12^.

Similar to other MO NPs, ZrO_2_ NPs are also key components of catalysts, dye-sensitized solar cells, and photodetectors^13–15^. There have been attempts to further enhance the catalytic activities of these NPs by doping with various metals (or anions) or modifying their surface morphologies via alteration of the number of defects such as oxygen vacancies and impurities^16,17^. In this study, our strategy to enhance the catalytic activities of ZrO_2_ NPs combined doping with Fe and increasing the surface structure deformation of the particles by varying the pH of the preparation condition; we named the resulting particles as Fe@ZrO_2_ NPs^18,19^. Fe ion is known to be naturally transported into and stored in cells and thereby used for intracellular physiological processes^20^; Fe is biocompatible. Therefore, the enhanced catalytic activities of the Fe@ZrO_2_ NPs may further extend their use to new applications in various fields including bio-related ones^21^.

However, NPs can cause also detrimental effects on environment, ultimately affecting human health, because of their potential toxicity to cells; therefore, their effects on the cell viability must be evaluated before use in applications especially involving contacts with human bodies^22^. We thus quantified the changes in the viability of human cells in the presence of Fe@ZrO_2_ NPs; in particular, we monitored the effects of NPs on the morphology, growth, metabolic actions, and intracellular organelle functions of human cell-lines that were developed from three organs or tissues that would be first or most affected by NPs (skin, lung, and kidney)^23–26^. We expected that our toxicity test results may support the potential of the Fe@ZrO_2_ NPs for use in not only conventional chemical reactions but also recently emerging biomedical and daily-life applications^27–30^.

After verifying that the developed Fe@ZrO_2_ NPs do not cause considerable detrimental effects on cell viability, we finally tested their catalytic activities for the oxidation of *L*-cysteine (Cys) (an example of biochemical reaction occurring in living bodies) by using high-resolution photoemission spectroscopy (HRPES). Cys is a small amino acid with a thiol group (-SH) that plays multiple important roles in cellular homeostasis, functions of proteins, and metabolism^31,32^. Therefore, the control of the state of Cys, either oxidized or reduced, is known to be important for biomedical systems^33,34^. In addition, we also assessed the photocatalytic activities of the four tested NPs for 4-chlorophenol (4-CP) degradation and OH radical formation of benzoic acid (BA) in aqueous solutions.

Fe@ZrO_2_ NPs that were prepared under a basic condition consistently exhibited the best catalytic activities for the different reactions. Our study demonstrates that if appropriately prepared, Fe@ZrO_2_ NPs can facilitate the control of reactions relevant to biomedical applications without severe negative effects on human cells.

## Results and Discussion

### Image-based analysis of cellular uptake of NPs

**Figure 1:**
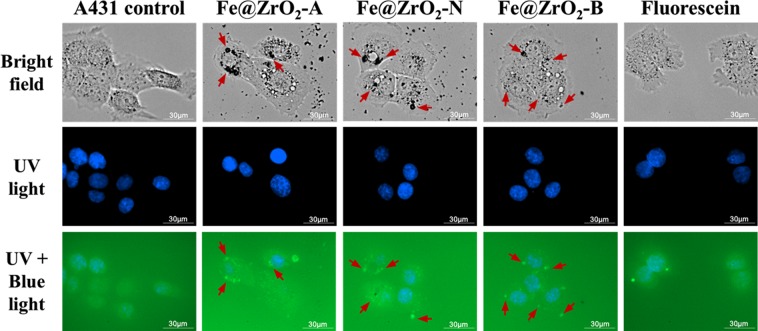
Microscopic images of A431 cells. The blue-colored areas are the cell nuclei. The arrows in the images indicate the intracellular loci of NPs.

Human cell uptake of NPs was observed using two cell-lines commonly chosen for toxicity assay, which had been originally isolated from the organs or tissues affected likely first and most by NPs: A431 cell line (from skin) and HEK 293T cell line (from kidneys)^35,36^. A431 cells treated with Fe@ZrO_2_ NPs were imaged using fluorescence microscopy. The NPs internalized into the cells were evident as black specks in the bright field images (Fig. 1). To better visualize the internalized NPs and their intracellular loci, the NPs were initially stained with a green fluorescein dye. The green dots in Fig. 1 indicate the internalized NPs; these appeared to be localized mostly in the peripheral cellular regions rather than in the central nucleus regions, which were specifically stained with Hoechst 33342 (blue-colored in the images) having affinity to DNA. The internalized NPs were consistently localized in the peripheral regions regardless of the pH variation in the NP preparation procedure. The loci of black specks in the bright field images matched well with the loci of green dots in the fluorescent images, verifying that NPs did not effectively penetrate into the nuclei of cells (the red arrows indicate the matched loci of NPs in the bright field and fluorescent images).

The genetic information that was encoded into the genomic DNA within the nucleus should be maintained well without considerable changes, for cells to keep growing. NPs have high activities for various physical and chemical processes; therefore, the NPs within the nucleus may cause alterations and even further breakage of genomic DNA^37^. Therefore, it is necessary to achieve minimal localization of NPs in the cell nucleus, as observed in our experiments, in order to guarantee the biosafety for use of NPs in applications.

In addition, time-lapse imaging of cells in the presence of NPs in culture media provided information on the dynamics of NP internalization into HEK 293T cells (Movies S1 and S2 in the electronic supplementary information (ESI)). Internalized NPs could be clearly recognized from 24 h post incubation of the cells with NPs. The particles were persistently kept inside the cells without apparent release into the culture media with the passage of time. However, as the cells divided into daughter cells, the number of NPs per cell gradually decreased (Movie S3 in the ESI). The imaging results also demonstrated that the internalized NPs were mostly localized in the peripheral region of the cells and that the presence of NPs did not significantly alter the cell morphology.

### Quantification of internalized NPs by flow cytometry

**Figure 2:**
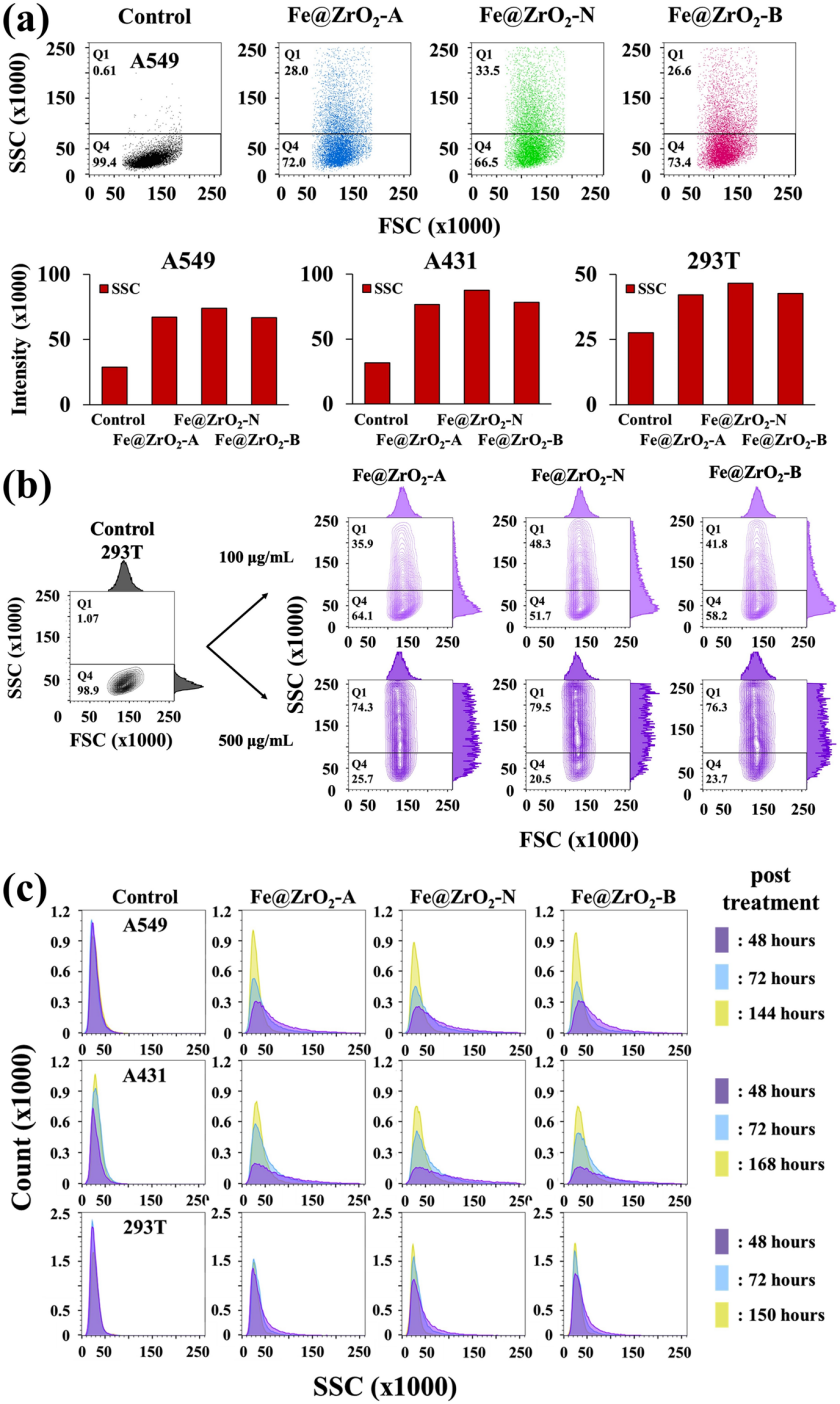
Flow cytometry analysis of cells with NPs inside. (**a**) Increase of side scatters by internalization of NPs (for A549, A431, and HEK 293T cells) (**b**) side scatter profiles of cell populations in the presence of NPs (for HEK 293T cells) (**c**) dynamic changes of side scatters for cells (for A549, A431, and HEK 293T cells).

To more quantitatively analyze the internalization of NPs into cells, we used flow cytometry. Flow cytometry analysis can provide quantitative information on the size (its increase is normally reflected by an increase in the forward scatters (FSCs) from cells, Figs 2 and 9) and internal complexity (its increase is reflected by an increase in the side scatters (SSCs) from cells) of cells^38,39^. While FSC from A549 cells did not change significantly with NP-treatment, SSC from the cells was apparently increased with the treatment (Fig. 2(a)), indicating that the internal complexity of the cells was increased by the internalization of NPs. The other two cell-lines, A431 and HEK 293T cells, also exhibited similar patterns during the flow cytometry analysis. In addition, all the variants of Fe@ZrO_2_ NPs, each prepared at different pH conditions (acidic, neutral, and basic), increased the internal complexity of the cells (via internalization) at equivalent levels (Fig. 2(a)).

**Figure 3:**
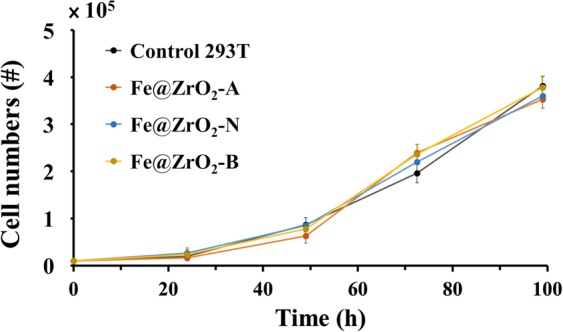
Growth dynamics of HEK 293T cells that were treated with NPs at the concentration of 100 μg/mL for 24 h.

**Figure 4:**
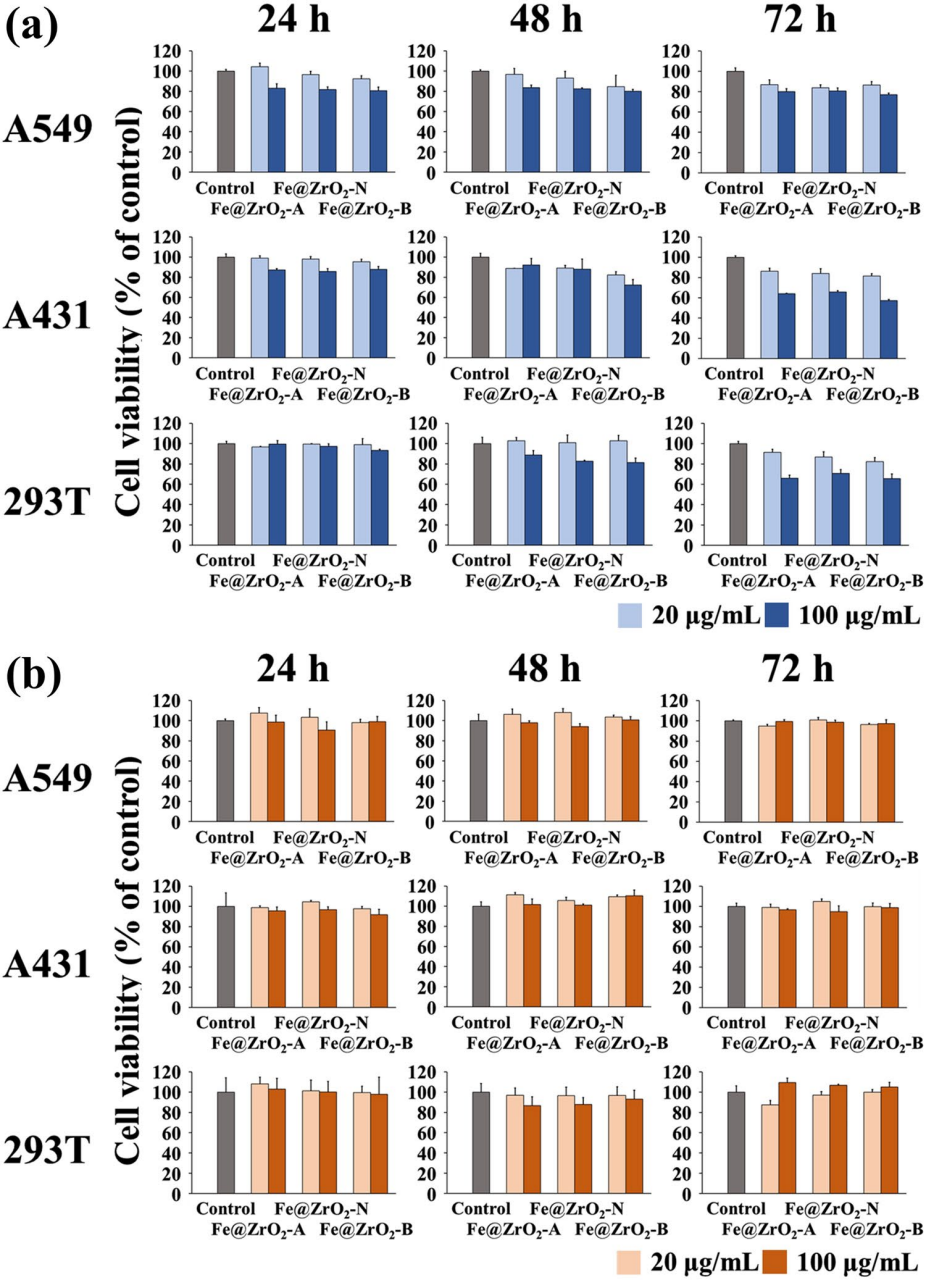
Quantification of cytotoxicity of NPs with MTT and NRU assays. A431, A549, and HEK 293T cells were treated with the Fe@ZrO_2_ variants at the concentrations of 20 and 100 μg/mL for 24–72 h. (**a**) MTT assay, (**b**) NRU assay.

**Figure 5:**
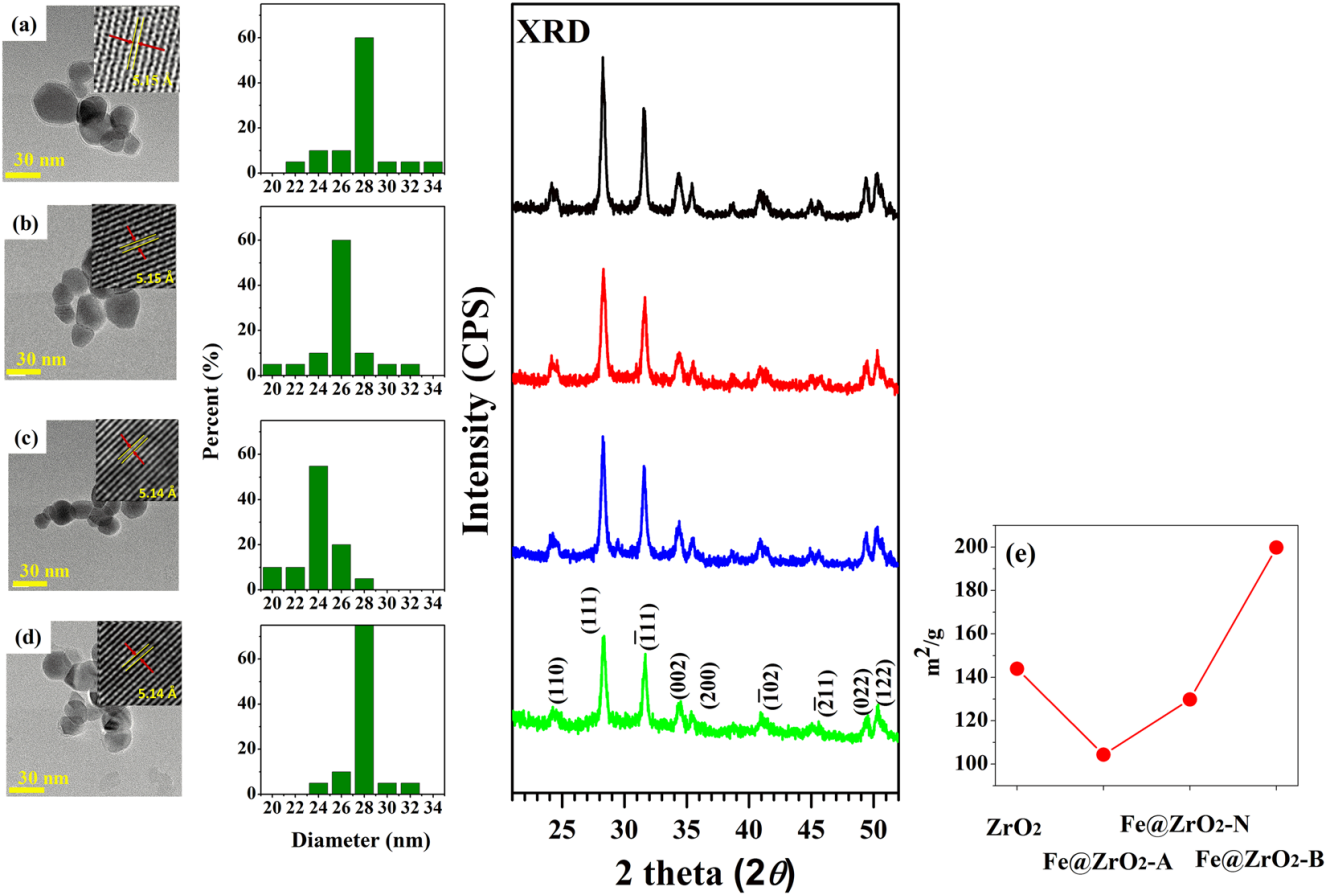
(Left) TEM images of the monoclinic 1 wt% ZrO_2_ NPs, (middle) their size distributions, and (right) their XRD spectra of (**a**) ZrO_2_, (**b**) Fe@ZrO_2_-A, (**c**) Fe@ZrO_2_-N, and (**d**) Fe@ZrO_2_-B. **(e**) BET results of 4 different NPs.

**Figure 6:**
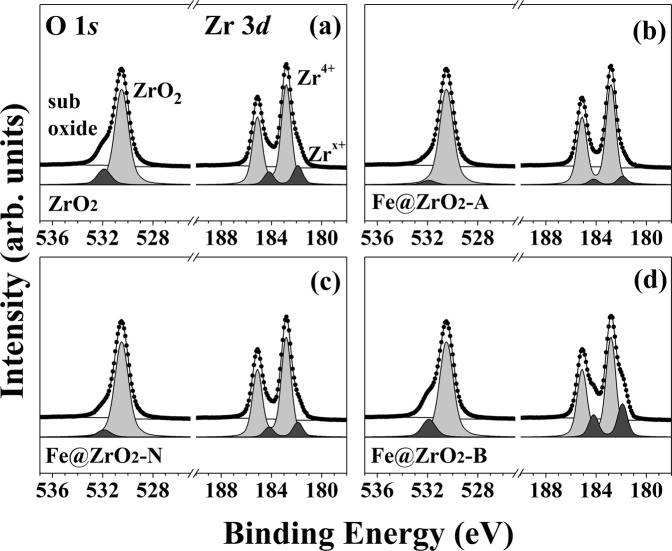
Zr 3*d* and O 1*s* core level spectra for (**a**) ZrO_2_ NPs, (**b**) Fe@ZrO_2_-A NPs, **(c**) Fe@ZrO_2_-N NPs, and (**d**) Fe@ZrO_2_-B NPs. Fe@ ZrO_2_-A, Fe@ ZrO_2_-N, and Fe@ ZrO_2_-B NPs indicate the ZrO_2_ NPs, Fe-doped ZrO_2_ NPs subjected to acid (pH = 1.5), neutral (pH = 7), or base (pH = 13.0) treatments.

**Figure 7:**
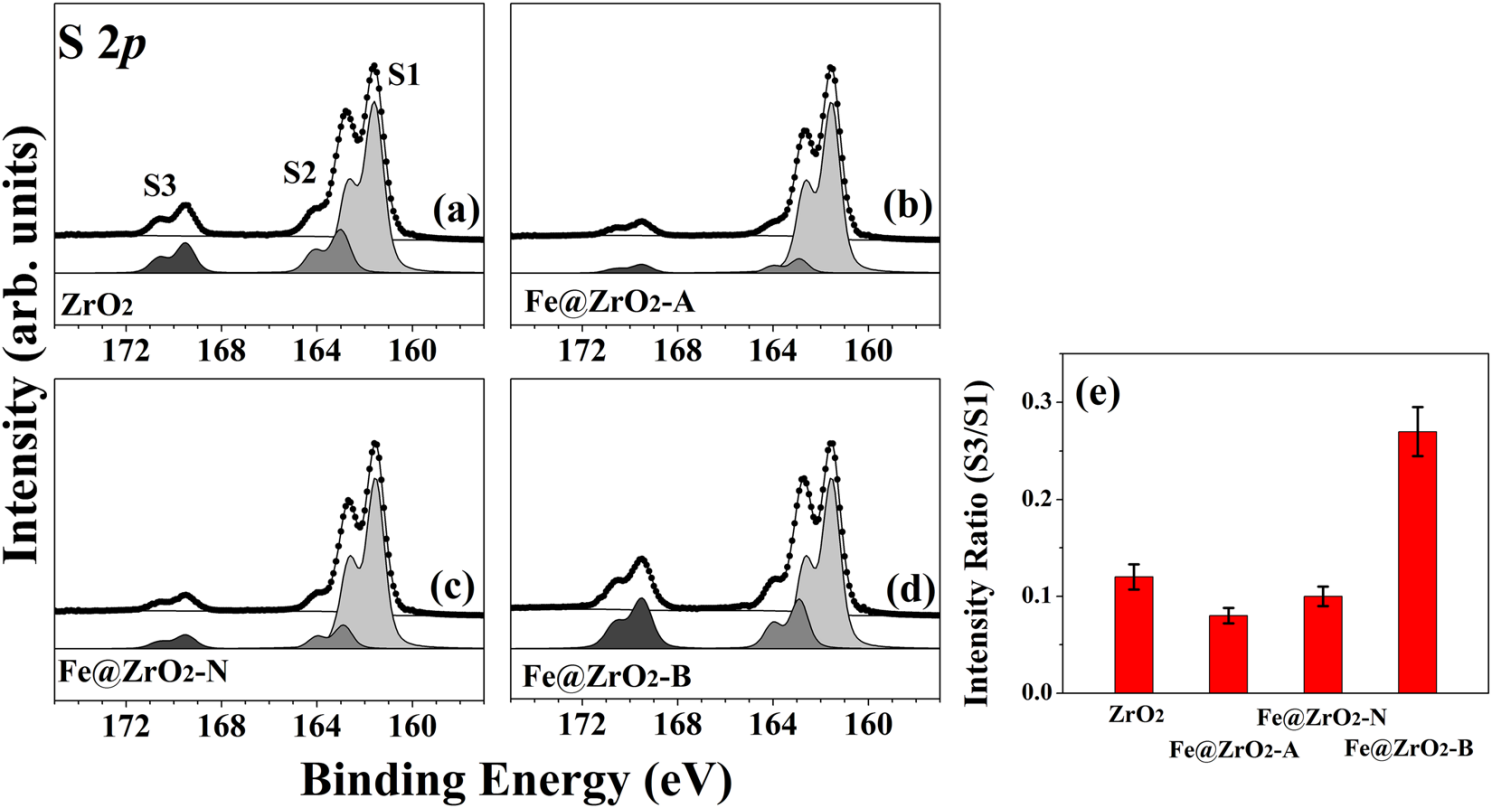
HRPES S 2*p* core-level spectra from the products of catalytic oxidations of cysteine (Cys, a 180 L solution) that were carried out with the following catalysts: (**a**) ZrO_2_ NPs, (**b**) Fe@ZrO_2_-A NPs, (**c**) Fe@ZrO_2_-N NPs, and (**d**) Fe@ZrO_2_-B NPs. (**e**) Values of the S3 to S1 ratio (see the text) for the four types of ZrO_2_ NPs, resulting from 180 L exposure of Cys solutions to UV light of wavelength 365 nm.

**Figure 8:**
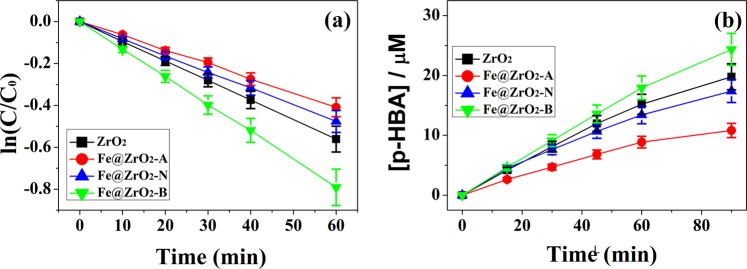
(**a**) 4-CP photocatalytic degradation and (**b**) Radical formation of *p*-HBA from BA reacted with four tested ZrO_2_ NPs. [TM@IrO_2_] = 0.4 g/L, [Benzoic acid]0 = 20 µM.

**Figure 9:**
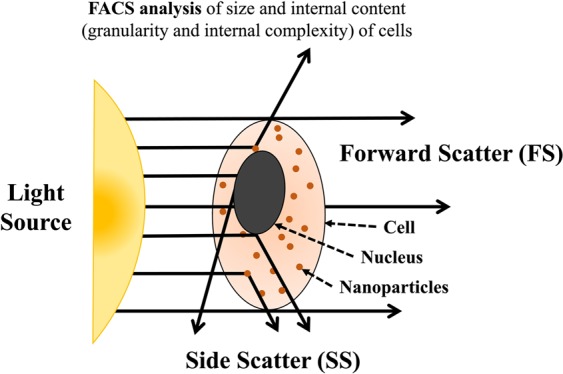
Flow cytometry analysis of cells.

In the presence of NPs at the concentration of 100 μg/mL, the percent of HEK 293T cells producing SSC above a base value (indicated by the lines in Fig. 2b) was increased to 35.9–41.8% from 1.95% of the control case. When treating cells with five-fold more NPs (i.e., at the concentration of 500 μg/mL), the percent of cells producing SSC above a base value was further increased to 74.3–79.5%. Responding to this concentration increase of NPs, HEK 293T cells also exhibited an increase in the population-averaged SSC intensities from 84,226–99,367 (for the NP treatment at 100 μg/mL) to 136,664–145,897 (for the NP treatment at 500 μg/mL). These observations indicate that as more NPs were provided into the environments surrounding cells, the cells take more NPs up. Flow cytometry measurements produced plausible positive correlations between the amount of supplied NPs and the cellular internal complexity, confirming the capability of flow cytometry to quantify the internalization of NPs into cells. Previously, it was revealed that the mean intensity of the SSCs from cells is proportional to the number of intracellular NPs^40,41^.

### Dynamic changes of intracellular NPs

To quantify the changes in the number of intracellular NPs as cells divided, flow cytometry analysis was performed for cells harboring NPs. A549, A431, and HEK 293T cells were treated with the three variants of Fe@ZrO_2_ NPs that had been prepared under acidic, neutral, and basic conditions at the concentration of 100 μg/mL. After 24 h incubation with NPs, the culture media were replaced with fresh ones, and the cells were further grown. At 48, 72, and over 144 h after NP-treatment, a fraction of the cells was sampled from the culture and analyzed using flow cytometry. With the passage of time and the accompanying cell division, the SSC from the cells gradually decreased, implying that the number of NPs per cell was reduced (Fig. 2(c)). For example, the population-averaged intensities of SSC at 72 h post NP-treatment were decreased approximately to the level equivalent to approximately 70% of the intensities obtained at 48 h post NP-treatment (A431 cell line). This decrease in the number of NPs per cell should mainly arise from the increase in the cell number as a result of divisions over time. Meanwhile, the release of NPs from the cells was not observed (Movie S4 in the ESI).

### Effects of NPs on the growth of cells

Intracellular NPs may negatively affect the growth of human cells^42,43^. To test this possibility, HEK 293T cells were first treated with the Fe@ZrO_2_ NP variants. At 24 h post NP-treatment, the culture media containing the NPs were replaced with fresh ones that were deficient in NPs; and the cells were then further cultured. As shown in Fig. 3, the growth rate of the HEK 293T cells that harbored NPs was similar to that of cells carrying no NP, implying that the Fe@ZrO_2_ NP variants do not negatively affect the growth of the cells. In addition, the growth dynamics of the cells harboring the NPs was similar, irrespective of the preparation pH-condition for the NP variants.

### Biochemical assessment of cell viability in the presence of NPs

Lack of negative effect of NPs on the growth of cells supports the potential of the NPs for uses in wide applications including daily-life and biomedical ones. To further test the biosafety of Fe@ZrO_2_ NPs, we employed biochemical cell viability assays. In these assays, cell viability is quantified by the metabolic activity of intracellular oxidoreductases (MTT assay) and the lysosomal uptake of Neutral Red (NRU assay), which are normally maintained at high levels for live eukaryotic cells^44^. MTT assay revealed that with NP-treatments at the concentrations of 20 and 100 μg/mL for 24 h, the three cell lines still had viability levels higher than 81% of the control case (without NP treatment, Fig. 4a). Longer incubations (48 h and 72 h) with NPs at the identical concentration range led to modest decrease of cell viability mostly to the levels around 80% of the control case or higher (though some cases showed decreases to the level of 72% and 57% of the control case for 48 h and 72 h incubations with the NPs at 100 μg/mL, respectively). Conventionally, MTT assay has been applied to quantify cell viability in the presence of NPs at the concentrations between 0.001 and 100 μg/mL^26,44^, which are lower than those of our experimental conditions. The presence of other types of MO NPs such as ZnO, TiO_2_, and previously studied ZrO_2_ at these lower concentrations caused significantly larger decreases in the MTT-based cell viability assay^45–48^. For example, ZnO and CuO NPs, which are often used in real-life applications, can decrease the viability of A549 cells to 30% of that of the control case. In others’ work, incubation for 24 h in the presence of NiO NPs at the concentration of 100 μg/mL decreased the viability of A549 cells to the level lower than 60% of that of the control, indicating that the NPs prepared in this study affect the cell viability to a lesser degree than those of previously developed NPs^49^.

The NRU assay results revealed that the lysosomal uptake of Neutral Red in cells pre-treated with NPs was maintained at the levels equivalent to that of the control case (without NP treatment). The cell viability of HEK 293T cells quantified by NRU uptake was only lowered by a maximum of 13% when treated with NPs (for the case of NP-treatment at the concentration of 100 μg/mL for 48 h, Fig. 4b). Quantifications of the two different modes of intracellular biochemical activities confirm that the developed variants of Fe@ZrO_2_ NPs exert only modest effects on the cell physiology and viability.

### Characterization of NPs

To assess the potential of the constructed NPs as catalysts, we preferentially analyzed the surface characters of those NPs. As mentioned in the Introduction section, one of the objectives of this study was to compare and analyze the variations in the photocatalytic properties of the Fe doped ZrO_2_ NPs that were prepared by chemical treatments at different pH conditions. TEM imaging revealed that the sizes of ZrO_2_ NPs and the three variants of Fe@ZrO_2_ NPs ranged from 20 nm to 34 nm in diameter with modest variations and those NPs had both round and irregular shapes (Fig. 5(a–d)). The four types of NP samples all generated XRD spectra with peaks at 2*θ* = 24.5°, 28.3°, 31.6°, 34.4°, 35.4°, 40.9°, 45.1°, 49.4°, and 50.5° corresponding to the characteristic reflections from the (110), (101), (−111), (002), (200), (−102), (−211), (022) and (122) planes of ZrO_2_. The TEM images and XRD patterns indicated the formation of single-phase ZrO_2_ NP, a result expected from successful Fe doping into the ZrO_2_ NPs without any phase transition^50,51^. In addition, for a structural perspective, the *c* axis lattice constants of the ZrO_2_, Fe@ZrO_2_-A, Fe@ZrO_2_-N, and Fe@ZrO_2_-B NPs were calculated by using the inverse fast Fourier transform (IFFT; inset images) and determined to be 5.15 ± 0.03, 5.15 ± 0.02, 5.14 ± 0.02, and 5.14 ± 0.02 Å, respectively. These highly similar lattice constants close to 5.14 Å indicate that the four NPs variants had nearly identical cubic structures^52^. This measurement result implies that Fe-doping does not cause structural changes of the ZrO_2_ NPs.

Next, the surface area of the NPs, as a property more directly affecting the performance of NPs as catalysts, was measured by BET experiments. In general, it is known that as the surface areas of NPs are increased, their catalytic activities can be also increased via adsorption of more reactant molecules. The average surface area of the Fe@ZrO_2_-B NPs (199.8 m^2^/g) was significantly higher than those of the ZrO_2_ (143.93 m^2^/g), Fe@ZrO_2_-A (104.3 m^2^/g), and Fe@ZrO_2_-N NPs (129.80 m^2^/g). The surface area of the Fe@ZrO_2_-B NPs is approximately 1.39 times higher than that of the ZrO_2_ NPs. Meanwhile, the surface area of the Fe@ZrO_2_-A NPs is 0.72 times less than that of the ZrO_2_ NPs. These differences in surface area are presumed to be related to the differences in the number of structural defects between those particles; more structural defects would increase the surface areas of the NPs^16,53^.

### Electronic structures of NPs

We also analyzed the bonding configuration of the surface Zr and O of the NP variants using HRPES shown in Fig. 6. The four ZrO_2_-containing samples (ZrO_2_, Fe@ZrO_2_-A, Fe@ZrO_2_-N, and Fe@ZrO_2_-B NPs) produced core level spectra, all containing two characteristic peaks that indicate the existence of defect structures within the NPs, but with different levels of intensities (Fig. 6); peaks corresponding to Zr 3*d*_5/2_ at 181.9 eV (Zr^x+^) and O 1 *s* at 531.8 eV (sub oxide)^54,55^. Interestingly, the spectra from Fe@ZrO_2_-B NPs had the highest intensities of the peaks for the states of Zr^x+^ and O sub-oxide. This result shows that Fe@ZrO_2_-B NPs had more defect structures compared with other variants of ZrO_2_ NPs, explaining, in part, why these NPs had larger surface areas than other variants (Fig. 5(e)). In addition, this experimental observation suggests that the portion of defect structures within NPs can be altered by chemical treatments involving pH variation.

### Oxidation activity for biochemical reaction

To assess the catalytic activities of the ZrO_2_ NPs and the three different Fe@ZrO_2_ NP variants, these NPs were employed to enhance an oxidation reaction of Cys molecules (180 L; saturation exposure: 1 L = 1 × 10^−6^ Torr/sec.). Three distinct 2*p*_3/2_ peaks in the HRPES S 2*p* core-level spectra were evident as shown in Fig. 7(a–d). The peaks at 161.5, 162.9, and 168.6 eV correspond to thiol group (-SH; denoted as S1), the bound state (denoted as S2), and sulfonic acid (SO_3_H) (denoted as S3), respectively. Since sulfonic acid is known to be an oxidation product of the thiol group^56,57^. The extent of oxidation of Cys can be quantified by the ratio of the intensities of the S1 and S3 peaks. The HRPES spectra data showed that Fe@ZrO_2_-B NPs could produce the oxidation product more than two-fold compared to other variants (Fig. 7(e)). This means that Fe@ZrO_2_-B NPs had the highest catalytic activity.

### Photocatalytic activities of NPs

Based on that Fe@ZrO_2_-B NPs oxidized Cys more effectively compared with other NPs, we were also curious of whether Fe@ZrO_2_-B NPs can show better photocatalytic activities than other particles. A general method for comparing the photocatalytic properties of MO NPs is to measure the degree of degradation of 4-chorolophenol (4-CP) in the presence of those particles^58^. As expected, time-course analysis also showed that the degradation rate of the reactant (4-CP) was higher in the presence of Fe@ZrO_2_–B NPs than the cases using other variants of NPs as catalysts (see Fig. 8(a)).

In detail, the apparent rate constant for the decay of the reactant was 1.31 × 10^−2^ min^−1^ when using Fe@ZrO_2_–B NPs, greater than the cases using other NPs (9.35 × 10^−3^ min^−1^ for ZrO_2_, 6.81 × 10^−3^ min^−1^ for Fe@ZrO_2_-A, and 7.93 × 10^−3^ min^−1^ for Fe@ZrO_2_-N NPs). In other word, the significantly higher rate constant for the degradation reaction in the presence of Fe@ZrO_2_–B NPs would be attributed to the higher surface area of the NPs compared with those of other particles. It has been previously reported that the formed electrons and holes can react with 4-CP in aqueous solution and subsequently produce ·OH radicals that are capable of effectively degrading the 4-CP^59,60^. Hence, to confirm that a large amount of ·OH radicals can be generated by action of the NPs as catalysts, a radical reaction of benzoic acid (BA) was performed.

$$\begin{array}{ccc}{{\rm{C}}}_{6}{{\rm{H}}}_{5}-{\rm{COOH}} & +\,\cdot {\rm{OH}}\,\,\mathop{\to }\limits^{hv} & {\rm{HO}}-{{\rm{C}}}_{6}{{\rm{H}}}_{5}-{\rm{COOH}}\\ {\rm{BA}} & {\mathrm{Fe}@\mathrm{ZrO}}_{2}\,{\rm{NPs}} & p \mbox{-} \mathrm{HBA}\end{array}$$

when the ·OH radical formed on the surface of NPs reacts with BA as shown in eqn. (1), *p*-hydroxy benzoic acid (*p*-HBA) is produced. From the time-course increase of the product we could compare the catalytic activities of the four differently prepared nanoparticles^61,62^.

As shown in Fig. 8(b), the four types of NPs had different levels of catalytic activities for the radical formation reaction. As we expected, Fe@ZrO_2_-B NPs exhibited better catalytic performance than other NPs did. Consistently high catalytic activities of Fe@ZrO_2_-B NPs for multiple reactions as shown above would imply that these NPs have properties generally advantageous for use as catalysts. We presume that more structural defects in Fe@ZrO_2_-B NPs, compared with other types of NPs, link to their higher surface areas and this difference in the physical character would explain the observed highest catalytic activities of the NPs for different types of reactions.

## Conclusion

We attempted to develop Fe@ZrO_2_ NPs with high catalytic activities but without any severe toxic effect. For this purpose, we prepared three different variants of the Fe@ZrO_2_ NPs by altering the pH condition of chemical treatment steps for the NPs (Fe@ZrO_2_-A, Fe@ZrO_2_-N, and Fe@ZrO_2_-B). We first confirmed that internalization of NPs of any kind did not considerably change morphology, growth rate, and biochemical activities of cells of different tissue origin. In contrast, Fe@ZrO_2_–B NPs showed highest catalytic activities among those NPs for multiple reactions. Therefore, our results proved that the concentration of defect structures (the formation of ·OH radical) is the most important factor that influences the quality of the catalyst, which can be enhanced by the pH treatment under basic conditions. Specifically, maximizing the number of defect structures while maintaining the characteristics of ZrO_2_ NPs could be the most effective way to improve the efficiency of the catalyst. The observed low level of toxicity and high level of catalytic activities support the potential of Fe@ZrO_2_ NPs in conventional chemical reactions as well as in recently emerging daily-life and biomedical applications. Conclusively, we believe that our approach of comparative analysis of catalytic properties after preferentially confirming cell toxicity for NPs will be extended the practical application industries using nanoparticles in the era of nanoscience and nano-engineering.

## Methods

### Observation and quantification of cellular uptake of NPs

To effectively monitor the cellular uptake of NPs, the NPs were stained with fluorescein dye. Fluorescein exhibits light excitation and emission peaks at 494 nm (observed as blue color) and 512 nm (observed as green color), respectively; therefore, fluorescein-stained NPs appear green. To perform real-time imaging of the cellular uptake of NPs, 1.5 × 10^4^ human skin cells (A431 cells) were first seeded in the wells of 24-well plates and cultured overnight. The cells were then incubated with fluorescein-stained Fe@ZrO_2_ NPs at the concentration of 100 μg/mL. After 24 h, the cells were washed twice with PBS, and the cell nuclei were stained, at 4 h post-washing, with Hoechst 33342 dye (blue, Thermo Fisher Scientific, Waltham, Massachusetts, USA) at the concentration of 10 ng/mL for 1 min in order to clearly identify the loci of the cells and to further distinguish the nuclear and peripheral regions of cells. After the nucleus staining, the cells were then washed with PBS. The cellular uptake of NPs was also monitored without fluorescein dye staining. All the real-time imaging of cells was performed using a live cell imaging system, Lionheart (BioTek, Winooski, Vermont, USA). To more systematically quantify the cellular uptake of NPs, the increase in the internal complexity of cells by the internalized NPs was measured using a flow cytometry system, FACSCantoTM II (BD Biosciences, San Jose, California, USA). Overall, forward scatter and side scatter signals were obtained from 30,000 cells, each of the two types of signals indicating the size and internal complexity, respectively, of the cells (see Fig. 9).

### Preparation of ZrO_2_ NPs

**Figure 10:**
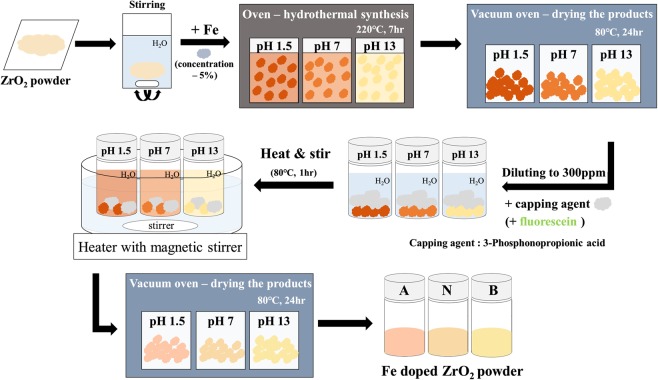
Procedure for preparing Fe@ZrO_2_ NPs.

Preparation of ZrO_2_ NPs and Fe-doping under different pH conditions. ZrO_2_ NPs were synthesized using a modified thermal method. Two precursor solutions, zirconium propoxide [Zr(OPr)_4_] in 2-propanol (~70 mM) and HNO_3_ in ethanol (~60 mM), were prepared; then, the HNO_3_ solution was gradually mixed into the Zr(OPr)_4_ solution with careful stirring for 1 h. The mixed solution of Zr(OPr)_4_ and HNO_3_ was next placed in an autoclave at 220 °C for 30 min and at 300 °C for 15 h. ZrO_2_ NPs were selectively precipitated in the mixed solution when cooled to room temperature, and the obtained NPs were then dried at 80 °C for 48 h. For doping ZrO_2_ NPs with Fe atom, precursor solutions were prepared using a one-pot synthesis. The desired amount of Fe dopants in the form of Fe(NO_3_)_3_∙9H_2_O (99% purity) was added with stirring into each synthetic gel solution to finally achieve Fe doping of 1 wt% based on the sum of Fe and ZrO_2_ NPs. The pH of each solution was then altered to the desired levels by using HNO_3_ (acid) and KOH (base). The solutions were transferred to a Teflon-lined autoclave and then sealed and heated at 220 °C for 7 h in a convection oven. The resulting ZrO_2_ and Fe@ZrO_2_ NPs (Fe@ZrO_2_-A, prepared with acid treatment (pH = 1.5); Fe@ZrO_2_-N, prepared with neutral treatment (pH = 7.0); and Fe@ZrO_2_-B, prepared with base treatment (pH = 13.0)) were filtered and washed with Deuterium-depleted water (DDW) to remove residues. In addition, to reduce the aggregation of NPs, the NPs were doped with 3-phosphonopropionic acid. This acid was introduced into the solutions of Fe@ZrO_2_ NPs during the doping step, and the resulting solutions were stirred at 80 °C for 1 h and then dried at 80 °C for 24 h. (see Fig. 10). All the substances were purchased from Sigma-Aldrich (St. Louis, Missouri, USA).

### Human cell culture

Human lung carcinoma epithelial cells (A549) and human skin epidermoid carcinoma cells (A431) were purchased from the Korean cell bank. These cells were cultured in RPMI 1640 medium (CORNING, Corning, NY, USA) supplemented with 10% Fetal Bovine Serum (FBS, Invitrogen, Carlsbad, CA, USA) and 1% penicillin-streptomycin (Pen Strep, Life Technologies, Carlsbad, CA, USA) at 37 °C and 5%-CO_2_ condition. Human embryonic kidney (HEK) 293T cells were cultured in Iscove’s modified Dulbecco’s medium (IMDM, Life Technologies) supplemented with 10% FBS and 1% Pen Strep at 37 °C and 5% CO_2_ condition.

### Introduction of Fe@ZrO_2_ NPs into cells

Base solutions containing Fe@ZrO_2_ NPs at the concentration of 100 mg/mL were prepared by mixing the synthesized Fe@ZrO_2_ NPs with UltraPure^TM^ Distilled Water (Invitrogen). The base solutions were then diluted further with distilled water to obtain solutions of lower NP-concentrations (2 and 10 mg/mL). Prior to introducing the NPs, the human cells were seeded and incubated in appropriate media (IMDM or RPMI 1640) for 24 h. The cells were seeded at similar numbers per surface area. Fe@ZrO_2_ NPs were then introduced into the cells in wells of 12-well, 24-well, or 96-well plates to attain the predetermined final NP-concentrations of 20 and 100 μg/mL in the culture media. The cells were further incubated in the presence of NPs for three different periods (24, 48, and 72 h). For the 72 h incubation case, the culture medium was replaced with a fresh one at 48 h after NP-introduction, to keep the cells growing. After the NP treatments, the cells were washed twice with phosphate-buffered saline (PBS, Gibco, Waltham, Massachusetts, USA). In addition, cells were also incubated in the absence of NPs for comparison.

### Quantification of growth of cells treated with NPs

In the wells of 12-well tissue culture plates, 3 × 10^4^ HEK 293T cells were seeded and incubated overnight. These cells were then treated with Fe@ZrO_2_-A, Fe@ZrO_2_-N, and Fe@ZrO_2_-B NPs at the concentration of 100 μg/mL. After 24 h, the NP-treated and untreated cells were washed twice with PBS. The cells with internalized NPs were then detached from the well surface with trypsin treatment, and 10^4^ cells were reseeded in the wells of 24-well plates. The cell growth dynamics was quantified by counting the cells in each well approximately every 24 h, with a hemocytometer.

### Quantification of the viability of cells in the presence of Fe@ZrO_2_ NPs

Two conventional assays, the MTT assay and the Neutral Red uptake (NRU) assay, were performed to quantify the cell viability in the presence of NPs. First, 3 × 10^3^ cells were seeded in the wells of 96-well tissue culture plates and cultured overnight. Fe@ZrO_2_ NPs were then introduced into the cultured cells at two concentrations (20 and 100 μg/mL), and the cells were further incubated for three different periods (24, 48, and 72 h) as steps of the viability tests.

For the MTT assay, 10 μL of the base MTT solution (5 mg/mL, M-5655, Sigma-Aldrich) was added to the culture medium covering the cells (the final MTT concentration in the medium was 500 μg/mL) at 24 h post NP-treatment. The cells were then incubated in the presence of MTT for 2 h (for the cells pre-treated with NPs for 48 and 72 h) or for 4 h (for the cells pre-treated with NPs for 24 h). The incubation time with MTT was varied according to the numbers of cells in the culturing wells. After the incubation period, 100 μL of MTT solubilization solution [M-8910, Sigma-Aldrich] was added to the culture media to dissolve the formed formazan crystals, of which amount indicates the metabolic activity of the mitochondria in living cells. The formed formazans were then quantified by measuring the absorbance of 570 nm light with a microplate reader (M5, Molecular Devices, San Jose, CA, USA). This MTT assay was performed using the *In Vitro* Toxicology Assay kit (Sigma Aldrich).

For the NRU assay, cells of the human kidney, skin, and lung (HEK 293T, A431, and A549 cells, respectively) were first treated with NPs. After 24 h, 5 μL or 10 μL of Neutral Red (NR) solution [N-2889, Sigma-Aldrich] was added to the culture media top of the cells; and the cells were then incubated at 37 °C and 5%-CO_2_ for 2 h (for HEK 293T cells) or 4 h (for A549 and A431 cells). After the incubation in the presence of NR, the cells were washed once with 50 μL of PBS or CaCl_2_ solution containing a fixative [N-4270, Sigma-Aldrich]. Next, 100 μL of Neutral Red Assay solubilization solution [N-4395] was added to the cells to dissolve the NR in the lysosomes. Finally, the absorption of light of wavelength 540 nm by the NR molecules within the cells was measured by using a microplate reader (M5). For this NRU assay, a kit from Sigma-Aldrich (*In Vitro* Toxicology Assay Kit – Neutral Red Based) was used.

### Characterization of Fe@ZrO_2_ NPs

The morphologies and sizes of ZrO_2_ and Fe@ZrO_2_ NPs were determined by using high-resolution transmission electron microscopy (HR-TEM, JEM-3010, JEOL Ltd, Japan) operating at 300 kV. Brunauer–Emmett–Teller (BET) surface area measurements were also performed for NPs by using an Autosorb-iQ 2ST/MP (Quanta chrome, Florida, USA). XRD patterns for NPs were obtained using Ni-filtered Cu–K*α* radiation from a Rigaku D/Max-A diffractometer (Matsubara-cho, Akishima-shi, Tokyo, JAPAN). High resolution photoemission spectroscopy (HRPES) experiments were performed at the 8A1 beamline of PAL with an electron analyzer (Physical Electronics, PHI-3057). The binding energies of the core level spectra were determined with respect to the binding energy (E_B_ = 84.0 eV) of the clean Au 4*f* core level for an identical photon energy. *L*-cysteine (HO_2_CCH(NH_2_)CH_2_SH, Sigma Aldrich, 99% purity) was purified by turbo pumping prior to dosing onto catalytic samples. A direct dozer with a variable leak valve was used to control the dose of the reactant molecules in reactions on NPs. The four types of Fe@ZrO_2_ NPs were irradiated with UV light (λ = 365 nm). The pressure of the chamber was maintained at 10^−6^ Torr during dosing, and the number of exposed molecules was defined by the dosing time in seconds (1 L = 1 × 10^−6^ Torr ·1 sec). 4-chlorophenol (30 mL/L) degradation and the radical formation of benzoic acid (20 µM) for the photocatalytic activity comparison of the four tested samples (0.4 g/L) were measured by using a 300-W Xe arc lamp (NEWPORT 300 W Xenon Light Source) with a 320 nm cut-off filter (for visible light irradiation).

## Supplementary information

MovieS4

MovieS3

MovieS2

MovieS1

## References

[1] Sikong L, Kongreong B, Kantachote D, Sutthisripok W (2010). Photocatalytic activity and antibacterial behavior of Fe^3+^-doped TiO_2_/SnO_2_ nanoparticles. Energy research journal.

[2] Ma Y (2014). Titanium dioxide-based nanomaterials for photocatalytic fuel generations. Chemical reviews.

[3] Li R (2015). Achieving overall water splitting using titanium dioxide-based photocatalysts of different phases. Energy & Environmental Science.

[4] Snaith HJ, Ducati C (2010). SnO_2_-based dye-sensitized hybrid solar cells exhibiting near unity absorbed photon-to-electron conversion efficiency. Nano letters.

[5] Zhang H (2012). Processing pathway dependence of amorphous silica nanoparticle toxicity: colloidal vs pyrolytic. J. Am. Chem. Soc..

[6] Xia T (2008). Comparison of the mechanism of toxicity of zinc oxide and cerium oxide nanoparticles based on dissolution and oxidative stress properties. ACS nano.

[7] Kaweeteerawat C (2015). Toxicity of metal oxide nanoparticles in Escherichia coli correlates with conduction band and hydration energies. Environmental science & technology.

[8] Yan X (2017). The interplay of sulfur doping and surface hydroxyl in band gap engineering: Mesoporous sulfur-doped TiO_2_ coupled with magnetite as a recyclable, efficient, visible light active photocatalyst for water purification. Applied Catalysis B: Environmental.

[9] Kustov A, Rasmussen SB, Fehrmann R, Simonsen P (2007). Activity and deactivation of sulphated TiO_2_-and ZrO_2_-based V, Cu, and Fe oxide catalysts for NO abatement in alkali containing flue gases. Applied Catalysis B: Environmental.

[10] Oh HS, Nong HN, Strasser P (2015). Preparation of mesoporous Sb‐, F‐, and In‐doped SnO_2_ bulk powder with high surface area for use as catalyst supports in electrolytic cells. Advanced Functional Materials.

[11] Yang G (2018). Effective carrier‐concentration tuning of SnO_2_ quantum dot electron‐selective layers for high‐performance planar perovskite solar cells. Advanced Materials.

[12] Tian ZR (2003). Complex and oriented ZnO nanostructures. Nature materials.

[13] Adamopoulos G (2011). High‐mobility low‐voltage ZnO and Li‐doped ZnO transistors based on ZrO_2_ high‐k dielectric grown by spray pyrolysis in ambient air. Advanced Materials.

[14] Haque MA, Sheikh AD, Guan X, Wu T (2017). Metal oxides as efficient charge transporters in perovskite solar cells. Advanced Energy Materials.

[15] Köck E-M, Kogler M, Bielz T, Klötzer B, Penner S (2013). *In situ* FT-IR spectroscopic study of CO_2_ and CO adsorption on Y_2_O_3_, ZrO_2_, and yttria-stabilized ZrO_2_. The Journal of Physical Chemistry C.

[16] Avadhut YS (2011). Study on the defect structure of SnO_2_: F nanoparticles by high-resolution solid-state NMR. Chemistry of Materials.

[17] Hwang YJ, Yang S, Lee H (2017). Surface analysis of N-doped TiO_2_ nanorods and their enhanced photocatalytic oxidation activity. Applied Catalysis B: Environmental.

[18] Hostaša J, Pabst W, Matějíček J (2011). Thermal conductivity of Al_2_O_3_–ZrO_2_ composite ceramics. J. Am. Cer. Soc..

[19] Sobańska K, Pietrzyk P, Sojka Z (2017). Generation of reactive oxygen species via electroprotic interaction of H_2_O_2_ with ZrO_2_ gel: ionic sponge effect and pH-switchable peroxidase-and catalase-like activity, *ACS*. Catalysis.

[20] Mackenzie EL, Iwasaki K, Tsuji Y (2008). Intracellular iron transport and storage: from molecular mechanisms to health implications. Antioxidants & redox signaling.

[21] Lunova M (2017). Nanoparticle core stability and surface functionalization drive the mTOR signaling pathway in hepatocellular cell lines. Sci. rep..

[22] Clift MJ, Gehr P, Rothen-Rutishauser B (2011). Nanotoxicology: a perspective and discussion of whether or not *in vitro* testing is a valid alternative. Archives of toxicology.

[23] Ivask A (2015). Toxicity of 11 metal oxide nanoparticles to three mammalian cell types *in vitro*. Current topics in medicinal chemistry.

[24] Sun T, Yan Y, Zhao Y, Guo F, Jiang C (2012). Copper oxide nanoparticles induce autophagic cell death in A549 cells. PLoS One.

[25] Choi S-J, Oh J-M, Choy J-H (2009). Toxicological effects of inorganic nanoparticles on human lung cancer A549 cells. Journal of Inorganic Biochemistry.

[26] Mahmoudi M, Laurent S, Shokrgozar MA, Hosseinkhani M (2011). Toxicity evaluations of superparamagnetic iron oxide nanoparticles: cell “vision” versus physicochemical properties of nanoparticles. ACS nano.

[27] Mahmoudi M, Hofmann H, Rothen-Rutishauser B, Petri-Fink A (2011). Assessing the *in vitro* and *in vivo* toxicity of superparamagnetic iron oxide nanoparticles. Chemical reviews.

[28] Lord H, Kelley SO (2009). Nanomaterials for ultrasensitive electrochemical nucleic acids biosensing. Journal of Materials Chemistry.

[29] Marchi J, Ussui V, Delfino CS, Bressiani AH, Marques MM (2010). Analysis *in vitro* of the cytotoxicity of potential implant materials. I: Zirconia‐titania sintered ceramics. Journal of Biomedical Materials Research Part B: Applied Biomaterials.

[30] Lunova M (2019). Targeting the mTOR Signaling Pathway Utilizing Nanoparticles: A Critical Overview. Cancers..

[31] Zhang G, Liu M (2000). Effect of particle size and dopant on properties of SnO_2_-based gas sensors. Sensors and Actuators B: Chemical.

[32] JungáKim M (2010). A thiol-specific fluorescent probe and its application for bioimaging. Chemical Communications.

[33] Wu S (2012). Selective electrochemical detection of cysteine in complex serum by graphene nanoribbon. Biosensors and Bioelectronics.

[34] Wang W (2005). Detection of homocysteine and cysteine. J. Am. Chem. Soc..

[35] Jain K (2014). *In vitro* cytotoxicity assessment of metal oxide nanoparticles. Nanomedicine and Nanobiology.

[36] Foldbjerg R, Dang DA, Autrup H (2011). Cytotoxicity and genotoxicity of silver nanoparticles in the human lung cancer cell line, A549. Archives of toxicology.

[37] Magdolenova Z (2014). Mechanisms of genotoxicity. A review of *in vitro* and *in vivo* studies with engineered nanoparticles. Nanotoxicology.

[38] Greulich C (2011). Uptake and intracellular distribution of silver nanoparticles in human mesenchymal stem cells. Acta biomaterialia.

[39] Suzuki H, Toyooka T, Ibuki Y (2007). Simple and easy method to evaluate uptake potential of nanoparticles in mammalian cells using a flow cytometric light scatter analysis. Environmental science & technology.

[40] Park J, Ha MK, Yang N, Yoon TH (2017). Flow cytometry-based quantification of cellular Au nanoparticles. Analytical chemistry.

[41] Zucker R, Massaro E, Sanders K, Degn L, Boyes W (2010). Detection of TiO_2_ nanoparticles in cells by flow cytometry. Cytometry Part A.

[42] Di Corato R (2012). Magnetic nanobeads decorated with silver nanoparticles as cytotoxic agents and photothermal probes. Small.

[43] Sreekanth T, Pandurangan M, Dillip G, Kim DH, Lee YR (2016). Toxicity and efficacy of CdO nanostructures on the MDCK and Caki-2 cells. Journal of Photochemistry and Photobiology B: Biology.

[44] Fotakis G, Timbrell JA (2006). *In vitro* cytotoxicity assays: comparison of LDH, neutral red, MTT and protein assay in hepatoma cell lines following exposure to cadmium chloride. Toxicology letters.

[45] Horie M (2012). Association of the physical and chemical properties and the cytotoxicity of metal oxide nanoparticles: metal ion release, adsorption ability and specific surface area. Metallomics.

[46] Karunakaran G (2013). Screening of *in vitro* cytotoxicity, antioxidant potential and bioactivity of nano-and micro-ZrO_2_ and-TiO_2_ particles. Ecotoxicology and environmental safety.

[47] Mftah A (2015). Physicochemical properties, cytotoxicity, and antimicrobial activity of sulphated zirconia nanoparticles. International journal of nanomedicine.

[48] Al-Fahdawi MQ (2015). Cytotoxicity and physicochemical characterization of iron–manganese-doped sulfated zirconia nanoparticles. International journal of nanomedicine.

[49] Mohamed K (2018). NiO nanoparticles induce cytotoxicity mediated through ROS generation and impairing the antioxidant defense in the human lung epithelial cells (A549): Preventive effect of Pistacia lentiscus essential oil. Toxicology Reports.

[50] Srinivasan R, De Angelis RJ, Ice G, Davis BH (1991). Identification of tetragonal and cubic structures of zirconia using synchrotron x-radiation source. J. Mater. Res..

[51] Reddy BM (2005). Structural characterization of nanosized CeO_2_-SiO_2_, CeO_2_-TiO_2_, and CeO_2_-ZrO_2_ catalysts by XRD, Raman, and HREM Techniques. J. Phys. Chem. B..

[52] Ploc R (1981). The lattice parameter of cubic ZrO_2_ formed on zirconium. Journal of Nuclear Materials.

[53] Deiana C, Fois E, Coluccia S, Martra G (2010). Surface structure of TiO_2_ P25 nanoparticles: Infrared study of hydroxy groups on coordinative defect sites. J. Phys. Chem. C..

[54] Jeong J, Lee B (2013). X-Ray photoelectron spectroscopy study of SnO_2_ and SnO_2 + x_ thin films,. J. Nanosci. Nanotechnol..

[55] Kim M-S (2004). Characteristics and processing effects of ZrO_2_ thin films grown by metal-organic molecular beam epitaxy. Appl. Surf. Sci..

[56] Meyers D (2013). Zhang-Rice physics and anomalous copper states in A-site ordered perovskites. Scientific Reports.

[57] Qiao R, Chin T, Harris SJ, Yan S, Yang W (2013). Spectroscopic fingerprints of valence and spin states in manganese oxides and fluorides. Current Applied Physics.

[58] Li X, Cubbage JW, Tetzlaff TA, Jenks WS (1999). Photocatalytic degradation of 4-chlorophenol. 1. the hydroquinone pathway. J. Org. Chem..

[59] Catalkaya EC, Bali U, Sengül F (2003). Photochemical degradation and mineralization of 4-chlorophenol. Environ. Sci. Pollut. Res. Int..

[60] Rajar K, Balouch SA, Bhanger MI, Sherazi TH, Kumar R (2018). Degradation of 4-chlorophenolunder sunlight using ZnO nanoparticles as catalysts. J. Electro. Mater..

[61] Wu C, De Visschera A, Gates ID (2017). Reactions of hydroxyl radicals with benzoic acid and benzoate. RSC Adv..

[62] Pang X (2014). Unraveling the photocatalytic mechanisms on TiO_2_ surfaces using the oxygen-18 isotopic label technique. molecules.

